# Supplementary material for: Identification of Freezing-Responsive microRNAs and Their Targets in Chinese Jujube by Small RNA and Degradome Sequencing
Source: Int J Mol Sci. 2026 Sep 4;27(17):7912. doi: 10.3390/ijms27177912 (PMC13566890; doi:10.3390/ijms27177912)
Supplement: Supplementary file 1 [file ijms-27-07912-s001.zip › Supplementary Material S2/novel_miR_216.pdf]

Provisional ID : novel_miR_216
Score total : 0.4
Score for star read(s) : -1.3
Score for read counts : 0
Score for mfe : 0.1
Score for randfold : 1.6
Score for cons. seed :
Total read count : 6639
Mature read count : 6638
Loop read count : 0
Star read count : 1

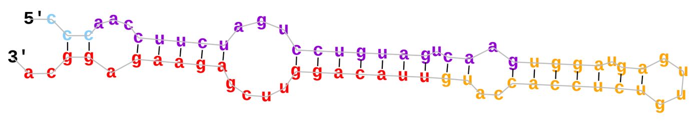

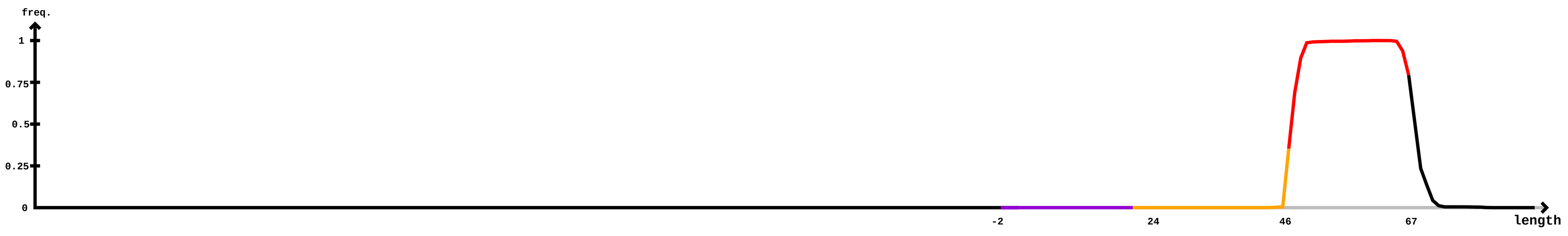

Star Mature

```
5'- aaggaguagucaaccaauuuaugccagacgccuccgauccaauuacaugugcuuuuaauggaauauccuauuaauuacacacacacuagaugccuaaccgaaaauuacuguagaauucauaauaaauuaauauauauaguacauauauuauaaugauagcccaaccuucuaguccuguagucaaguggaugaguugucuccaccauguuacagguucgagaagaggcaggcacaaacaaacauauauuau -3' obs
    aaggaguagucaaccaauuuaugccagacgccuccgauccaauuacaugugcuuuuaauggaauauccuauuaauuacacacacacuagaugccuaaccgaaaauuacuguagaauucauaauaaauuaauauauauaguacauauauuauaaugauagcccaaccuucuaguccuguagucaaguggaugaguugucuccaccauguuacagguucgagaagaggcaggcacaaacaaacauauauuau     exp
    ..((((..(((...((.....))...)))..))))............((((...(((((((......)))))))...)))).........(((((..........(((((((.....................)))))))(((........)))...(((....(((((...(((((((.((.(((((.((....)))))))..)))))))))....))))).))))))))..................   reads  mm  sample
    ..................................................................................................................................................................aaccuucuaguccuguagucaag.................................................................   1  0  JCK-1
    ............................................................................................................................................................................................................auCuuacagguucgagaag...........................   1  1  LT-3
    ............................................................................................................................................................................................................auCuuacagguucgagaagagg........................   1  1  LCK-1
    ............................................................................................................................................................................................................auCuuacagguucgagaagaggc.......................   1  1  JT-3
    ............................................................................................................................................................................................................auNuuacagguucgagaagaggcagg....................   1  1  LT-2
    ............................................................................................................................................................................................................auCuuacagguucgagaagaggcaggc...................   1  1  JCK-2
    .............................................................................................................................................................................................................uCuuacagguucgagaagag.........................   1  1  LT-2
    .............................................................................................................................................................................................................uCuuacagguucgagaagagg........................   1  1  LCK-1
    .............................................................................................................................................................................................................uCuuacagguucgagaagagg........................   2  1  LT-2
    .............................................................................................................................................................................................................uCuuacagguucgagaagagg........................   1  1  LCK-3
    .............................................................................................................................................................................................................uCuuacagguucgagaagaggc.......................   1  1  LT-3
    .............................................................................................................................................................................................................uCuuacagguucgagaagaggca......................   1  1  LCK-3
    .............................................................................................................................................................................................................uCuuacagguucgagaagaggca......................   2  1  JT-3
    .............................................................................................................................................................................................................uCuuacagguucgagaagaggca......................   2  1  LT-2
    .............................................................................................................................................................................................................uCuuacagguucgagaagaggcaggc...................   1  1  LT-2
    ..............................................................................................................................................................................................................Cuuacagguucgagaaga..........................   1  1  LCK-1
    ..............................................................................................................................................................................................................Cuuacagguucgagaagagg........................   2  1  LT-2
    ..............................................................................................................................................................................................................Uuuacagguucgagaagaggca......................   6  1  LT-2
    ..............................................................................................................................................................................................................Cuuacagguucgagaagaggca......................   1  1  LT-1
    ..............................................................................................................................................................................................................guuacagguucgagaagaggca......................   1  0  LCK-3
    ..............................................................................................................................................................................................................Cuuacagguucgagaagaggcag.....................   1  1  LCK-3
    ..............................................................................................................................................................................................................Cuuacagguucgagaagaggcagg....................   1  1  JT-1
    ..............................................................................................................................................................................................................Cuuacagguucgagaagaggcaggc...................   1  1  LCK-3
    ...............................................................................................................................................................................................................uuacagguucgagaagag.........................   9  0  JT-2
    ...............................................................................................................................................................................................................uuacagguucgagaagag.........................   1  0  LT-3
    ...............................................................................................................................................................................................................uuacagguucgagaagag.........................   6  0  LT-1
    ...............................................................................................................................................................................................................uuacagguucgagaagag.........................   2  0  LCK-3
    ...............................................................................................................................................................................................................uuacagguucgagaagag.........................   7  0  JT-3
    ...............................................................................................................................................................................................................uuacagguucgagaagag.........................   5  0  LT-2
    ...............................................................................................................................................................................................................uuacagguucgagaagagg........................   6  0  JCK-3
    ...............................................................................................................................................................................................................uuacagguucgagaagagg........................   35 0  LT-3
    ...............................................................................................................................................................................................................uuacagguucgagaagagg........................   3  0  JCK-2
    ...............................................................................................................................................................................................................uuacagguucgagaagagg........................   4  0  JCK-1
```

Star **Mature**

aaggaguagucaaccaauuuaugccagacgccuccgauccaauuacaugugcuuuuaauggaauauccuauuaauuacacacacacuagaugccuaaccgaaaauuacuguagaauucauaauaaauuaauauauauaguacauauauuauaaugauagcccaaccuucuaguccuguagucaaguggaugaguugucuccaccauguuacagguucgagaagaggcaggcacaaacaaacauauauuau

uuacagguucgagaagagg	30	0	JT-2
uuacCgguucgagaagagg	1	1	LT-3
uuacagguucgagaagagg	21	0	LT-1
uuacagguucgagaagagU	1	1	LT-2
uuacagguucgagaagagg	16	0	LT-2
uuacagguucgagaagagg	8	0	LCK-3
uuacCgguucgagaagagg	1	1	LCK-3
uuacagguucgagaagagA	9	1	LCK-3
uuacagguucgagaagagg	44	0	JT-3
uuacagguucgagaagaggc	66	0	LT-3
uuacagguucgagaagaggc	25	0	LCK-3
uuacagguucgagaagaggc	57	0	LT-2
uuacagguucgagaagaggc	13	0	JT-3
uuacagguucgagaagaggc	69	0	LT-1
uuacCgguucgagaagaggc	1	1	LT-3
uuNcagguucgagaagaggc	1	1	LT-2
uuacagguucgagaagaggc	7	0	JCK-2
uuacagguucgagaagaggc	12	0	JT-2
uuacagguucgagaagaggc	11	0	JT-1
uuacagguucgCgaagaggc	1	1	LT-2
uuacagguucgagaagaggc	6	0	JCK-1
uuacagguucgagaagaggc	1	0	JCK-3
uuacCgguucgagaagaggca	1	1	LCK-3
uuacagguucgagaagaggca	13	0	JCK-2
Cuacagguucgagaagaggca	1	1	LCK-3
uuacagguucgagaagaAgca	1	1	LT-1
uuacaAguucgagaagaggca	1	1	LT-2
uuacagguucgagaagaggca	15	0	JCK-1
uuacagguucgCgaagaggca	1	1	LT-2
uuacagguucgCgaagaggca	1	1	LCK-2
uuacagguucgagaagaggca	83	0	LCK-1
uuacagguucgagaagaggca	4	0	JCK-3
uuacagguucgagaagaggca	159	0	LT-3
uuacagguucgagaagaggca	243	0	LT-2
uuacagguucgagaagaggca	414	0	LCK-3
uuacagguucgagaagaggca	44	0	JT-2
uuacagAuucgagaagaggca	1	1	LCK-3
uuacCgguucgagaagaggca	1	1	LT-2
uuacagguucgagaagaggca	23	0	JT-3
uuacagguucgagaagagAca	1	1	LT-3
uuaAagguucgagaagaggca	1	1	LT-1
uNacagguucgagaagaggca	1	1	JT-2
uuacagguucgagaagaggUa	1	1	JCK-3
uuacagguucgagaagaggca	70	0	LCK-2
uuNcagguucgagaagaggca	1	1	LT-2
uuacagguucgagaagaggca	161	0	LT-1
uuacagguucgagaagaggcag	26	0	LT-2
uuacagguucgagaagaggcag	16	0	LT-3
uuacagguucgagaagaggcag	28	0	LCK-1
uuacagguucgagaagaggcag	14	0	LT-1
uuacagguucgagaagaggcag	43	0	LCK-3
uuacagguucgagaagaggcag	26	0	JT-3
uuacagguucgagaagaggcag	17	0	JT-2
uuacCgguucgagaagaggcag	1	1	LT-3
uuacagguucgagaagaggcag	23	0	LCK-2
uuacagguucgagaagaggcagg	6	0	JT-1
uuacagguucgagaagaggcagg	44	0	LT-1
uuacagguucgagaagaggcagg	16	0	JT-3
uuacagguucgagaagaggcagg	5	0	JCK-2
uuacagguucgagaagaggcagg	2	0	JCK-1
uuacagguucgagaagaggcagg	145	0	LCK-3
uuacagguucgagaagaggcagU	3	1	LT-2
uuacagguucgagaagaggcagg	2	0	JCK-3
uuacagguucgagaagaggcagg	28	0	LT-3
uuacagguucgagaagaggcagg	37	0	LT-2
uuacagguucgagaagaggcagg	12	0	JT-2
uuacagguucgagaagaggcagA	1	1	LT-1
uuacagguucgagaaUaggcaggc	1	1	JCK-1
uuacagguucgagaagaggcaggc	17	0	LT-3
uuacagguucgagaagaggcaggc	17	0	LT-1

Star **Mature**

aaggaguagucaaccaauuuaugccagacgccuccgauccaauuacaugugcuuuuaauggaauauccuauuaauuacacacacacuagaugccuaaccgaaaauuacuguagaauucauaauaaauuaauauauauaguacauauauuauaaugauagcccaaccuucuaguccuguagucaaguggaugaguugucuccaccauguuacagguucgagaagaggcaggcacaaacaaacauauauuau

uuacagguucgagaaUaggcaggc	1	1	JCK-2
uuacagguucgagaagaggcaggc	16	0	LT-2
uuacagguucgagaagaggcaggc	2	0	JCK-3
uuacagguucgagaagaggcaggc	10	0	LCK-3
uuacagguucgagaagaggcaggc	1	0	JCK-2
uuacagguucgagaagaggcaggU	1	1	LT-3
uuacagguucgagaaUaggcaggc	3	1	JCK-3
uuacagguucgagaagaggcaggca	2	0	JT-2
uuacagguucgagaagaggcaggca	4	0	LT-1
uuacagguucgagaagaggcaggca	3	0	LT-2
uuacagguucgagaagaggcaggca	14	0	LCK-3
uuacagguucgagaagaggcaggca	2	0	JT-3
uuacagguucgagaagaggcaggca	5	0	LT-3
uuacagguucgagaagaggcaggca	2	0	JCK-1
uuacagguucgCgaagaggcaggca	1	1	LCK-3
uuacagguucgagaagaggcaggca	3	0	JCK-2
uuacagguucgagaagaggcaggcac	1	0	LT-1
uuacagguucgagaagaggcaggcac	2	0	LT-2
uuacagguucgagaagaggcaggcac	3	0	JCK-2
uacagguucgagaagagg	1	0	JT-1
uacagguucgagaagagg	51	0	LT-2
uacagguucgagaagagg	51	0	LT-1
uacCgguucgagaagagg	1	1	LT-3
uacagguucgagaagagg	7	0	JCK-2
uacagguucgagaagagg	11	0	JT-3
uacagguucgagaagagg	28	0	LCK-3
uacagguucgagaagagg	37	0	LT-3
uacagguucgagaagagg	3	0	JCK-3
uacagguucgagaagagg	5	0	JT-2
uacagguucgagaagagg	3	0	JCK-1
uacCgguucgagaagagg	1	1	LT-2
uacagguucgagaagaggc	83	0	LT-1
uacagguucgagaagaggc	9	0	JCK-2
uacagguucgagaagaggc	77	0	LT-3
uacagguucgagaagaggc	76	0	LCK-3
uacagguucgagaagaggc	5	0	JT-3
uacagguucgagaagaggc	77	0	LT-2
uacCgguucgagaagaggc	1	1	LCK-2
uacagguucgagaagaggc	1	0	JCK-1
uacagguucgagaagaggc	3	0	JCK-3
uacagguucgagaagaggc	24	0	LCK-1
uacagguucgagaagaggc	4	0	JT-2
uacagguucgagaagaggc	22	0	LCK-2
uacCgguucgagaagaggc	1	1	LT-2
uacagguucgagaagaggca	47	0	LCK-1
uacagguucgagaagaggca	24	0	JT-2
uacagguucgagaagaggca	129	0	LT-1
uacagguucgagaagaggca	44	0	LCK-2
uacagguucgagaagaggca	13	0	JT-3
uacagguucgagaagaggca	250	0	LCK-3
uacagguucgagaagaggca	184	0	LT-2
uacagguucgagaagaggca	155	0	LT-3
uacagguucgagaagaggca	6	0	JCK-3
uacagguucgagaagaggca	39	0	JT-1
uacagguucgCgaagaggca	1	1	LT-2
uacagguucgagaagaggca	10	0	JCK-1
uacCgguucgagaagaggca	1	1	LCK-3
uacCgguucgagaagaggca	2	1	LT-2
uacCgguucgagaagaggca	1	1	LT-3
uacagguucgagaagaggca	11	0	JCK-2
uacagguucgCgaagaggcag	1	1	LCK-3
uacagguucgagaagaggcag	65	0	LCK-3
uacagguucgagaagaggcag	19	0	LT-3
uacagguucgagaagaggcag	26	0	LCK-2
uacagguucgagaagaggcag	25	0	LT-2
uacCgguucgagaagaggcag	1	1	LCK-3
uacagguucgagaagaggcag	22	0	LT-1
uacagguucgagaagaggcag	2	0	JT-3
uacagguucgagaCgaggcag	1	1	LCK-1
uacagguucgagaagaggcag	2	0	JT-2

```
                                                                                                                                                                                         Star                                                  Mature
aaggaguagucaaccaauuuaugccagacgccuccgauccaauuacaugugcuuuuaauggaauauccuauuaauuacacacacacuagaugccuaaccgaaaauuacuguagaauucauaauaaauuaauauauauaguacauauauuauaaugauagcccaaccuucuaguccuguagucaaguggaugaguugucuccaccauguuacagguucgagaagaggcaggcacaaacaaacauauauuau
................................................................................................................................................................................................................uacagguucgagaagaggUag.....................	6	1	JT-2
................................................................................................................................................................................................................uacagguucgagaagaggcag.....................	22	0	LCK-1
................................................................................................................................................................................................................uacagguucgagaagaggcag.....................	1	0	JCK-2
................................................................................................................................................................................................................uacagguucgagaagaggcag.....................	2	0	JCK-3
................................................................................................................................................................................................................uacagguucgagaagaggUag.....................	3	1	JT-3
................................................................................................................................................................................................................uacagguucgagaagaggcag.....................	1	0	JCK-1
................................................................................................................................................................................................................uacagguucgagaagaggcagg....................	17	0	LT-3
................................................................................................................................................................................................................uacagguucgagaagaggcagg....................	20	0	LT-1
................................................................................................................................................................................................................uacagguucgagaagaggcagg....................	2	0	JCK-3
................................................................................................................................................................................................................uacagguucgagaagaggcagg....................	34	0	LT-2
................................................................................................................................................................................................................uacagguucgagaagaggcagg....................	51	0	LCK-3
................................................................................................................................................................................................................uacagguucgagaagaggcagg....................	2	0	JT-3
................................................................................................................................................................................................................uacagguucgagaagaggcagg....................	2	0	JCK-2
................................................................................................................................................................................................................uacagguucgagaagaggcagg....................	1	0	JCK-1
................................................................................................................................................................................................................uacagguucgagaagaggcagA....................	2	1	LT-3
................................................................................................................................................................................................................uacagguucgagaaNaggcaggc...................	1	1	LCK-1
................................................................................................................................................................................................................uacagguucgagaagaggcaggc...................	24	0	LT-3
................................................................................................................................................................................................................uacagguucgagaagaggcaggA...................	1	1	LT-3
................................................................................................................................................................................................................uacagguucgagaagaggcaggc...................	32	0	LT-2
................................................................................................................................................................................................................uacagguucgagaagaggcaggc...................	58	0	LCK-1
................................................................................................................................................................................................................uacagguucgagaagaggcaggc...................	54	0	LCK-3
................................................................................................................................................................................................................uacagguucgagaagaggcaggU...................	2	1	JCK-1
................................................................................................................................................................................................................uacagguucgagaagaggcaggc...................	3	0	JCK-1
................................................................................................................................................................................................................uacagguucgagaagaggcaggc...................	15	0	JCK-2
................................................................................................................................................................................................................uacagguucgagaagaggcaggc...................	27	0	LT-1
................................................................................................................................................................................................................uacagguucgagaagaggcaggc...................	5	0	JCK-3
................................................................................................................................................................................................................uacagguucgagaNgaggcaggc...................	1	1	LCK-1
................................................................................................................................................................................................................uacagguucgagaagaggcaggc...................	69	0	LCK-2
................................................................................................................................................................................................................uacagguucgagaagaggcagUc...................	1	1	LCK-2
................................................................................................................................................................................................................uacagguucgagaagaggcaggca..................	6	0	JT-3
................................................................................................................................................................................................................uacagguucgagaagaggcaggca..................	1	0	JCK-3
................................................................................................................................................................................................................uacagguucgagaagaggcaggca..................	4	0	LT-3
................................................................................................................................................................................................................uacagguucgagaagaggcaggca..................	35	0	LCK-2
................................................................................................................................................................................................................uacagguucgagaagaggcaggca..................	5	0	LT-1
................................................................................................................................................................................................................uacagguucgagaagaggcaggca..................	1	0	JT-2
................................................................................................................................................................................................................uacagguucgagaagaggcaggca..................	28	0	LCK-1
................................................................................................................................................................................................................uacagguucgagaagaggcaggca..................	1	0	JCK-2
................................................................................................................................................................................................................uacagguucgagaagaggcaggca..................	10	0	LT-2
................................................................................................................................................................................................................uacagguucgagaagaggcaggcac.................	1	0	LT-3
................................................................................................................................................................................................................uacagguucgagaagaggcaggcac.................	9	0	LT-1
................................................................................................................................................................................................................uacagguucgagaagaggcaggcac.................	9	0	LCK-3
................................................................................................................................................................................................................uacagguucgagaagaggcaggcac.................	3	0	LT-2
.................................................................................................................................................................................................................acagguucgagaagaggc.......................	7	0	JCK-2
.................................................................................................................................................................................................................acagguucgagaagaggc.......................	98	0	LT-3
.................................................................................................................................................................................................................acagguucgagaagaggc.......................	61	0	LT-1
.................................................................................................................................................................................................................acagguucgagaagaggc.......................	84	0	LT-2
.................................................................................................................................................................................................................acagguucgagaagaggc.......................	5	0	JCK-3
.................................................................................................................................................................................................................acagguucgagaagaggc.......................	12	0	JT-1
.................................................................................................................................................................................................................acagguucgagaagaggc.......................	2	0	JT-3
.................................................................................................................................................................................................................acagguucgagaagaggc.......................	41	0	LCK-3
.................................................................................................................................................................................................................acagguucgagaagaggc.......................	1	0	JT-2
.................................................................................................................................................................................................................acagguucgagaagaggc.......................	1	0	JCK-1
.................................................................................................................................................................................................................acagguucgCgaagaggca......................	1	1	LCK-2
.................................................................................................................................................................................................................acagguucgagaagaggca......................	123	0	LT-2
.................................................................................................................................................................................................................acagguucgagaagaggca......................	79	0	LT-1
.................................................................................................................................................................................................................acagguucgagaagaggca......................	5	0	JCK-1
.................................................................................................................................................................................................................acNgguucgagaagaggca......................	1	1	LT-2
.................................................................................................................................................................................................................acagguucgagaagaggca......................	3	0	JCK-3
.................................................................................................................................................................................................................acCgguucgagaagaggca......................	3	1	LT-2
.................................................................................................................................................................................................................acagguucgagaagaggca......................	79	0	LCK-1
.................................................................................................................................................................................................................acagguucgagaagaggca......................	8	0	JCK-2
.................................................................................................................................................................................................................aGagguucgagaagaggca......................	1	1	LT-2
.................................................................................................................................................................................................................acagguucgagaagaggcU......................	1	1	LT-3
.................................................................................................................................................................................................................acagguucgagaagaggca......................	122	0	LCK-3
.................................................................................................................................................................................................................acagguucgagaagaggca......................	99	0	LT-3
.................................................................................................................................................................................................................acagguucgagaagaggca......................	14	0	JT-2
.................................................................................................................................................................................................................acagguucgagaagaggca......................	16	0	JT-3
.................................................................................................................................................................................................................acagguucgagaagaggca......................	85	0	LCK-2
.................................................................................................................................................................................................................acagguucgagaagaggcag.....................	13	0	LT-2
.................................................................................................................................................................................................................acagguucgagaagaggcag.....................	13	0	JT-1
```

Star Mature

```
aaggaguagucaaccaauuuaugccagacgccuccgauccaauuacaugugcuuuuaauggaauauccuauuaauuacacacacacuagaugccuaaccgaaaauuacuguagaauucauaauaaauuaauauauauaguacauauauuauaaugauagcccaaccuucuaguccuguagucaaguggaugaguugucuccaccauguuacagguucgagaagaggcaggcacaaacaaacauauauuau
.................................................................................................................................................................................................................acagguucgagaagaggcag.....................    17    0    LT-3
.................................................................................................................................................................................................................acagguucgagaagaggcag.....................    3     0    JCK-2
.................................................................................................................................................................................................................acagguucgagaagaggcag.....................    1     0    JCK-3
.................................................................................................................................................................................................................acagguucgagaagaggcag.....................    8     0    JT-3
.................................................................................................................................................................................................................acagguucgagaagaggcag.....................    7     0    JT-2
.................................................................................................................................................................................................................acagguucgagaagaggcag.....................    14    0    LT-1
.................................................................................................................................................................................................................acagguucgagaagaggcag.....................    41    0    LCK-3
.................................................................................................................................................................................................................acagguucgagaagaggcag.....................    5     0    JCK-1
.................................................................................................................................................................................................................acagguucgagaagaggcagg....................    4     0    JCK-2
.................................................................................................................................................................................................................acagguucgagaagaggcagg....................    1     0    JCK-1
.................................................................................................................................................................................................................acagguucgagaagaggcagg....................    43    0    LCK-3
.................................................................................................................................................................................................................acagguucgagaagaggcagg....................    33    0    LT-1
.................................................................................................................................................................................................................acagguucgagaagaggcagg....................    27    0    LT-2
.................................................................................................................................................................................................................acagguucgagaagaggcagg....................    8     0    LT-3
.................................................................................................................................................................................................................acagguucgagaagaggcaAg....................    1     1    LCK-3
.................................................................................................................................................................................................................acagguucgagaagaggcagg....................    4     0    JT-3
.................................................................................................................................................................................................................acagguucgagaagaggcagg....................    4     0    JT-2
.................................................................................................................................................................................................................acagguucgagaagaggcaggc...................    44    0    LCK-1
.................................................................................................................................................................................................................acagguucgagaagaggcaggc...................    16    0    LT-2
.................................................................................................................................................................................................................acagguucgagaagaggcaggc...................    14    0    LCK-3
.................................................................................................................................................................................................................acagguucgagaagaggcaggc...................    51    0    LCK-2
.................................................................................................................................................................................................................acagguucgagaagaggcaggc...................    13    0    LT-3
.................................................................................................................................................................................................................acagguucgagaagaggcaggc...................    19    0    LT-1
.................................................................................................................................................................................................................acagguucgagaagaggcaggca..................    1     0    LT-2
.................................................................................................................................................................................................................acagguucgagaagaggcaggca..................    7     0    LT-3
.................................................................................................................................................................................................................acagguucgagaagaggcaggca..................    6     0    LT-1
.................................................................................................................................................................................................................acagguucgagaagaggcaggcac.................    2     0    JCK-3
.................................................................................................................................................................................................................acagguucgagaagaggcaggcac.................    2     0    LT-1
.................................................................................................................................................................................................................acagguucgagaagaggcaggcac.................    5     0    LT-2
.................................................................................................................................................................................................................Ucagguucgagaagaggcaggcacaaaca............    3     1    JT-3
..................................................................................................................................................................................................................cagguucgagaagaggca......................    14    0    JT-3
..................................................................................................................................................................................................................cagguucgagaagaggca......................    59    0    LCK-2
..................................................................................................................................................................................................................cagguucgagaagaggca......................    54    0    LT-2
..................................................................................................................................................................................................................cagguucgagaagaggca......................    34    0    LT-1
..................................................................................................................................................................................................................cCgguucgagaagaggca......................    1     1    LT-2
..................................................................................................................................................................................................................cagguucgagaagaggca......................    48    0    LT-3
..................................................................................................................................................................................................................cagguucgagaagaNgca......................    1     1    LCK-1
..................................................................................................................................................................................................................cagguucgagaagaggca......................    18    0    JT-2
..................................................................................................................................................................................................................cagguucgagaagaggca......................    1     0    JCK-2
..................................................................................................................................................................................................................cagguucgagaagaggca......................    22    0    LCK-3
..................................................................................................................................................................................................................cagguucgagaagaggca......................    73    0    LCK-1
..................................................................................................................................................................................................................cagguucgagaCgaggca......................    1     1    LT-2
..................................................................................................................................................................................................................cCgguucgagaagaggca......................    1     1    LT-1
..................................................................................................................................................................................................................cagguucgagaagaggca......................    2     0    JCK-1
..................................................................................................................................................................................................................cagguucgagaagaggca......................    2     0    JCK-3
..................................................................................................................................................................................................................cagguucgagaagaggcag.....................    8     0    LCK-3
..................................................................................................................................................................................................................cagguucgagaagaggcag.....................    11    0    LT-2
..................................................................................................................................................................................................................cagguucgagaagaggcag.....................    19    0    JT-3
..................................................................................................................................................................................................................cagguucgagaagaggcag.....................    3     0    LT-3
..................................................................................................................................................................................................................cagguucgagaagaggcag.....................    13    0    JT-2
..................................................................................................................................................................................................................cagguucgagaagaggcag.....................    1     0    JCK-2
..................................................................................................................................................................................................................cagguucgagaagaggcag.....................    6     0    LT-1
..................................................................................................................................................................................................................cagguucgagaagaggcagg....................    16    0    LT-3
..................................................................................................................................................................................................................cagguucgagaagaggcagg....................    5     0    LT-2
..................................................................................................................................................................................................................cagguucgagaagaggcagg....................    10    0    JT-2
..................................................................................................................................................................................................................cagguucgagaagaggcagg....................    2     0    JCK-2
..................................................................................................................................................................................................................cagguucgagaagaggcagg....................    9     0    LT-1
..................................................................................................................................................................................................................cagguucgagaagaggcagg....................    16    0    JT-3
..................................................................................................................................................................................................................cagguucgagaagaggcagA....................    1     1    LT-2
..................................................................................................................................................................................................................cagguucgagaagaggcagg....................    1     0    JCK-1
..................................................................................................................................................................................................................cagguucgagaagaggcagg....................    9     0    LCK-3
..................................................................................................................................................................................................................cagguucgagaagaggcaggc...................    1     0    JCK-2
..................................................................................................................................................................................................................cagguucgagaagaggcaggc...................    5     0    LT-1
..................................................................................................................................................................................................................cagguucgagaagaNgcaggc...................    1     1    LCK-1
..................................................................................................................................................................................................................cagguucgagaagaggcaggc...................    15    0    LCK-3
..................................................................................................................................................................................................................cagguucgagaagaggcaggc...................    28    0    LCK-2
..................................................................................................................................................................................................................cagguucgagaagaggcaggc...................    2     0    LT-2
..................................................................................................................................................................................................................cagguucgagaagaggcaggc...................    26    0    LCK-1
..................................................................................................................................................................................................................cagguucgagaagaggcaggc...................    6     0    LT-3
..................................................................................................................................................................................................................cagguucgagaagaggcaggca..................    1     0    LT-1
```

Star Mature

```
aaggaguagucaaccaauuuaugccagacgccuccgauccaauuacaugugcuuuuaauggaauauccuauuaauuacacacacacuagaugccuaaccgaaaauuacuguagaauucauaauaaauuaauauauauaguacauauuauaaugauagcccaaccuucuaguccuguagucaaguggaugaguugucuccaccauguuacagguucgagaagaggcaggcacaaacaaacauauauuau
................................................................................................................................................................................................................cagguucgagaagaAgcaggca..................   1   1   LT-3
................................................................................................................................................................................................................cagguucgagaagaggcaggca..................   1   0   JT-2
................................................................................................................................................................................................................cagguucgagaagaggcaggca..................   7   0   JT-3
................................................................................................................................................................................................................cagguucgagaagaggcaggca..................   12  0   LCK-1
................................................................................................................................................................................................................cagguucgagaagaggcaggca..................   2   0   LT-3
................................................................................................................................................................................................................cagguucgagaagaggcaggca..................   1   0   JCK-3
................................................................................................................................................................................................................cagguucgagaagaggcaggca..................   31  0   LCK-2
................................................................................................................................................................................................................cagguucgagaagaggcaggcac.................   2   0   JCK-1
................................................................................................................................................................................................................cagguucgagaagaggcaggcac.................   2   0   LT-1
................................................................................................................................................................................................................cagguucgagaagaggcaggcac.................   3   0   JCK-2
................................................................................................................................................................................................................cagguucgagaagaggcaggcac.................   1   0   LT-2
.................................................................................................................................................................................................................agguucgagaagaggcag.....................   1   0   LT-1
.................................................................................................................................................................................................................agguucgagaagaggcag.....................   2   0   LCK-3
.................................................................................................................................................................................................................agguucgagaagaggcagg....................   1   0   LT-1
.................................................................................................................................................................................................................agguucgagaagaggcagg....................   8   0   JT-3
.................................................................................................................................................................................................................agguucgagaagaggcagg....................   3   0   LT-2
.................................................................................................................................................................................................................agguucgagaagaggcagg....................   7   0   LT-3
.................................................................................................................................................................................................................agguucgagaagaggcagg....................   3   0   JT-2
.................................................................................................................................................................................................................agguucgagaagaggcaggc...................   2   0   LT-3
.................................................................................................................................................................................................................agguucgagaagaggcaggc...................   2   0   LT-2
.................................................................................................................................................................................................................agguucgagaagaggcaggc...................   2   0   LT-1
.................................................................................................................................................................................................................agguucgagaagaggcaggc...................   1   0   JCK-2
.................................................................................................................................................................................................................agguucgagaagaggcaggca..................   2   0   LT-3
.................................................................................................................................................................................................................agguucgagaagaggcaggcac.................   1   0   LT-1
..................................................................................................................................................................................................................gguucgagaagaggcagg....................   5   0   LCK-3
..................................................................................................................................................................................................................gguucgagaagaggcaggc...................   2   0   LT-2
..................................................................................................................................................................................................................gguucgagaagaggcaggc...................   1   0   LT-1
..................................................................................................................................................................................................................gguucgagaCgaggcaggc...................   1   1   LT-2
..................................................................................................................................................................................................................gguucgagaagaggcaggca..................   1   0   LT-1
...................................................................................................................................................................................................................guucgagaagaggcaggc...................   1   0   LT-1
...................................................................................................................................................................................................................guucgagaagaggcaggc...................   1   0   LT-3
...................................................................................................................................................................................................................guucgagaagaggcaggca..................   3   0   JT-3
...................................................................................................................................................................................................................guucgagaagaggcaggca..................   1   0   JT-2
....................................................................................................................................................................................................................uucgagaagaggcaggca..................   4   0   JT-2
....................................................................................................................................................................................................................uucgagaagaggcaggca..................   2   0   LT-3
....................................................................................................................................................................................................................uucgagaagaggcaggca..................   3   0   JT-3
....................................................................................................................................................................................................................uucgagaagaggcaggcac.................   2   0   LT-2
......................................................................................................................................................................................................................cgagaagaggcaggcacaaa..............   1   0   JCK-2
.......................................................................................................................................................................................................................gagaagaggcaggcacaaaca............   1   0   JCK-3
.......................................................................................................................................................................................................................gagaagaggcaggcacaaaca............   1   0   JCK-2
.......................................................................................................................................................................................................................gagaagaggcaggcacaaacaaaU.........   1   1   JCK-2
.......................................................................................................................................................................................................................gagaagaggcaggcacaaacaaaU.........   2   1   JCK-1
.......................................................................................................................................................................................................................gagaagaggcaggcacaaacaaaU.........   1   1   JCK-3
........................................................................................................................................................................................................................agaagaggcaggcacaaac.............   1   0   JCK-3
........................................................................................................................................................................................................................agaagaggcaggcacaaacaa...........   2   0   JT-2
........................................................................................................................................................................................................................agaagaggcaggcacaaacaa...........   1   0   JT-3
........................................................................................................................................................................................................................agaagaggcaggcacaaacaaa..........   3   0   JT-2
........................................................................................................................................................................................................................agaagaggcaggcacaaacaaa..........   4   0   JT-3
..........................................................................................................................................................................................................................aagaggcaggcacaaaca............   1   0   JCK-1
..........................................................................................................................................................................................................................aagaggcaggcacaaacaaU..........   1   1   JCK-2
...........................................................................................................................................................................................................................agaggcaggcacaaacaaa..........   4   0   JT-3
...........................................................................................................................................................................................................................agaggcaggcacaaacaaa..........   4   0   JT-2
```
